# Spatio-temporal ultrasound beam modulation to sequentially achieve multiple foci with a single planar monofocal lens

**DOI:** 10.1038/s41598-021-92849-x

**Published:** 2021-06-29

**Authors:** Sergio Pérez-López, José Miguel Fuster, Pilar Candelas

**Affiliations:** Centro de Tecnologías Físicas, Universitat Politècnica de València, 46022 Valencia, Spain

**Keywords:** Physics, Applied physics, Acoustics

## Abstract

Ultrasound focusing is a hot topic due to its multiple applications in many fields, including biomedical imaging, thermal ablation of cancerous tissues, and non destructive testing in industrial environments. In such applications, the ability to control the focal distance of the ultrasound device in real-time is a key advantage over conventional devices with fixed focal parameters. Here, we present a method to achieve multiple time-modulated ultrasound foci using a single planar monofocal Fresnel Zone Plate. The method takes advantage of the focal distance linear dependence on the operating frequency of this kind of lenses to design a sequence of contiguous modulated rectangular pulses that achieve different focal distances and intensities as a function of time. Both numerical simulations and experimental results are presented, demonstrating the feasibility and potential of this technique.

## Introduction

Focusing ultrasonic waves has multiple applications in various fields, such as non-destructive testing in industrial scenarios^1^, biomedical imaging of different kind of tissues^2^, or thermal ablation of tumours through High Intensity Focused Ultrasound (HIFU)^3–5^. Different techniques have been devised in the literature to achieve multiple acoustic foci. Among all the possible options, one of the main and most used methods is employing phased arrays, which allows to generate different acoustic foci by adjusting the time delay of each one of its different transducer elements^6–8^. A more recent approach to achieve arbitrary pressure fields consists of using acoustic holograms directly coupled to a single ultrasound transducer, which provides a simpler and cheaper, yet very powerful and versatile method to provide complex 3D pressure distributions^9–14^. However, in contrast to phased arrays, acoustic holograms do not allow to dynamically control the ultrasound beam once the lens is manufactured.

In this work, we present a spatio-temporal beam modulation technique to achieve multiple foci using a conventional Fresnel Zone Plate (FZP). With this method, an arbitrary number of foci and relative acoustic intensities can be multiplexed in the time domain, achieving, therefore, an ultrasonic focal beam that can be controlled in both space and time.

FZPs are widely used monofocal planar lenses made of a series of concentric rings, known as Fresnel regions, with decreasing width. Due to their easy design, manufacturing process, and focusing capabilities, this kind of devices are employed in a wide range of fields ranging from optics^15–17^ to X-ray^18,19^, microwaves^20,21^, and acoustics^22–26^. Moreover, in the past years novel designs based on the FZP structure have been presented, which allow the formation of acoustic vortex^27^, and bifocal^28,29^ and fractal^30,31^ intensity distributions, increasing the versatility and interest of this type of lenses.

For a FZP lens, the diffracted pressure waves at two consecutive Fresnel regions reach the focus with a complex phase difference of $\pi$ (in phase opposition), meaning that those pressure waves interfere destructively. This $\pi$ phase change results in a propagation path difference of $\lambda /2$ between consecutive radii, which directly yields to the design equation of the lens:

1 $$\begin{aligned} d+F+\frac{n\lambda }{2} = \sqrt{d^2+r_n^2} + \sqrt{F^2+r_n^2}, \end{aligned}$$

where *d* is the distance between transducer and lens, *F* is the focal distance, $\lambda$ is the working wavelength, $r_n$ is the *n*th radius of the lens, and $n=1,2,\ldots ,N$, being *N* the total number of Fresnel regions. Two kinds of FZPs can be distinguished depending on the physical implementation of the phase-opposition regions. Thus, Soret FZPs alternate pressure blocking with transparent regions^22,23,26^, while Phase-Reversal FZPs replace blocking areas with phase inverting regions^24,32^.

## Results

One intrinsic property of FZPs is that they are designed to operate at a single frequency, as the different radii of the lens depend on the central design wavelength. When the frequency is reduced or increased from the central design frequency, there is a phase error among the pressure contributions of the different regions that yields to a focal shift from its theoretical focal distance. This focal shift results in undesired chromatic aberration in optical imaging scenarios^15^, but in acoustics it can actually be used as a dynamic focal control method^33^. In fact, recently, researchers have introduced focal tunability to this kind of devices by shifting the operating frequency to control the focal distance of an airborne acoustic vortex generated by an active-spiral FZP^34^, or by designing a stretchable silicon FZP^35^.

**Figure 1:**
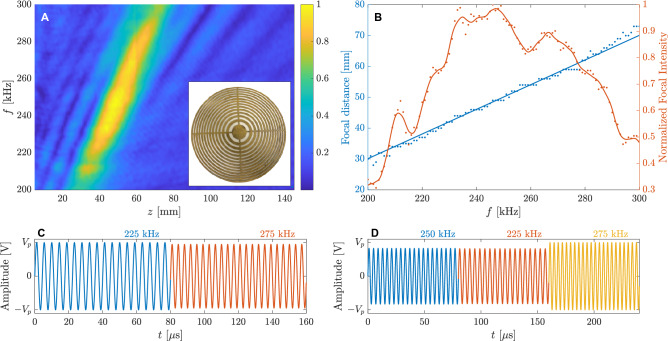
Time-modulation design technique: (**A**) measured axial spectrum of the Fresnel Zone Plate lens and (**B**) focal distance (left) and normalized focal intensity (right) as a function of the operating frequency. (**C**) Waveform designed to achieve two equal intensity foci at 40 and 60 mm and (**D**) waveform designed to achieve three foci at 50, 40, and 60 mm with normalized intensities 0.8, 0.6, and 1, respectively. In (**B**), solid dots correspond to experimental measurements while solid lines correspond to a linear fit to the data (blue) and a Savitzky–Golay interpolation filter (red).

Operating the FZP at frequencies around the central design frequency of the lens yields to a linear shift in the focus position with low distortion in terms of resolution and focal shape. One important parameter that can be used to characterize the frequency response of the lens is its axial spectrum, which represents the absolute value of the pressure profile (i.e., the pressure distribution along the central axis of the lens) as a function of the frequency. In this sense, Fig. 1A represents the measured axial spectrum of a Soret FZP made of brass immersed in a distilled water tank, designed for $d=350$ mm, $F=50$ mm, $f=250$ kHz (which results in $\lambda =6$ mm considering a speed of sound $c_0=1500$ m/s in water), and $N=27$. The lens was manufactured in brass due to its high impedance mismatch with water ($Z_{brass}=40$ MRayls compared to $Z_{water}=1.5$ MRayls), which ensures a good pressure blocking performance. However, despite this high impedance mismatch, a brass plate immersed in water presents a resonant behaviour with full transmission coefficient at frequencies where the brass thickness is an integer multiple of $\lambda _{brass}/2$, which means that the thickness of the lens has to be selected so that the resonant frequencies are far away from the operating frequency range. In this case, the manufactured lens has a maximum radius of 104.4 mm and a thickness of $t_h=1$ mm, which provides a first resonant mode at 2.35 MHz. The measurements were carried out using an automated 3D scanning system with a 1 mm needle hydrophone from Precision Acoustics Ltd. The signal was generated using an Arbitrary Waveform Generator (AWG) connected to a 75 W power amplifier and an Imasonic 250 kHz piston transducer with an aperture of 30 mm (see “Supplementary Information” for more details on the axial spectrum measurement process). It is worth noting that the axial spectrum is influenced by the frequency response of the transducer, which affects the amplitude of the transmitted pressure depending on the frequency of the input waveform.

Due to the planar geometry of the FZP, the system will present a transient response^36^, as the contributions of the different Fresnel regions will reach the focus at different times due to the distance difference in their propagation paths. Thus, the steady state is reached when the pressure waves diffracted at the different regions of the lens overlap at the focus simultaneously. In this case, the transient state duration ($\Delta t$) can be calculated as the difference between the time of arrival of the pressure wave diffracted at the first Fresnel region (shortest propagation path) and the time of arrival of the pressure wave diffracted at the last Fresnel region (longest propagation path), that is,

2 $$\begin{aligned} \Delta t = \frac{ \left( \sqrt{d^2+r_{27}^2}+\sqrt{F^2+r_{27}^2}\right) - \left( \sqrt{d^2+r_{1}^2}+\sqrt{F^2+r_{1}^2}\right) }{c_0}. \end{aligned}$$

This transient state duration represents the maximum response speed of the system. It is worth noting that the transient state duration only depends on the geometric properties of the system, and not on the operating frequency. The transient state duration for the 40, 50, and 60 mm foci are 55.62, 52.00, and 48.72 $\upmu$s, respectively.

**Figure 2:**
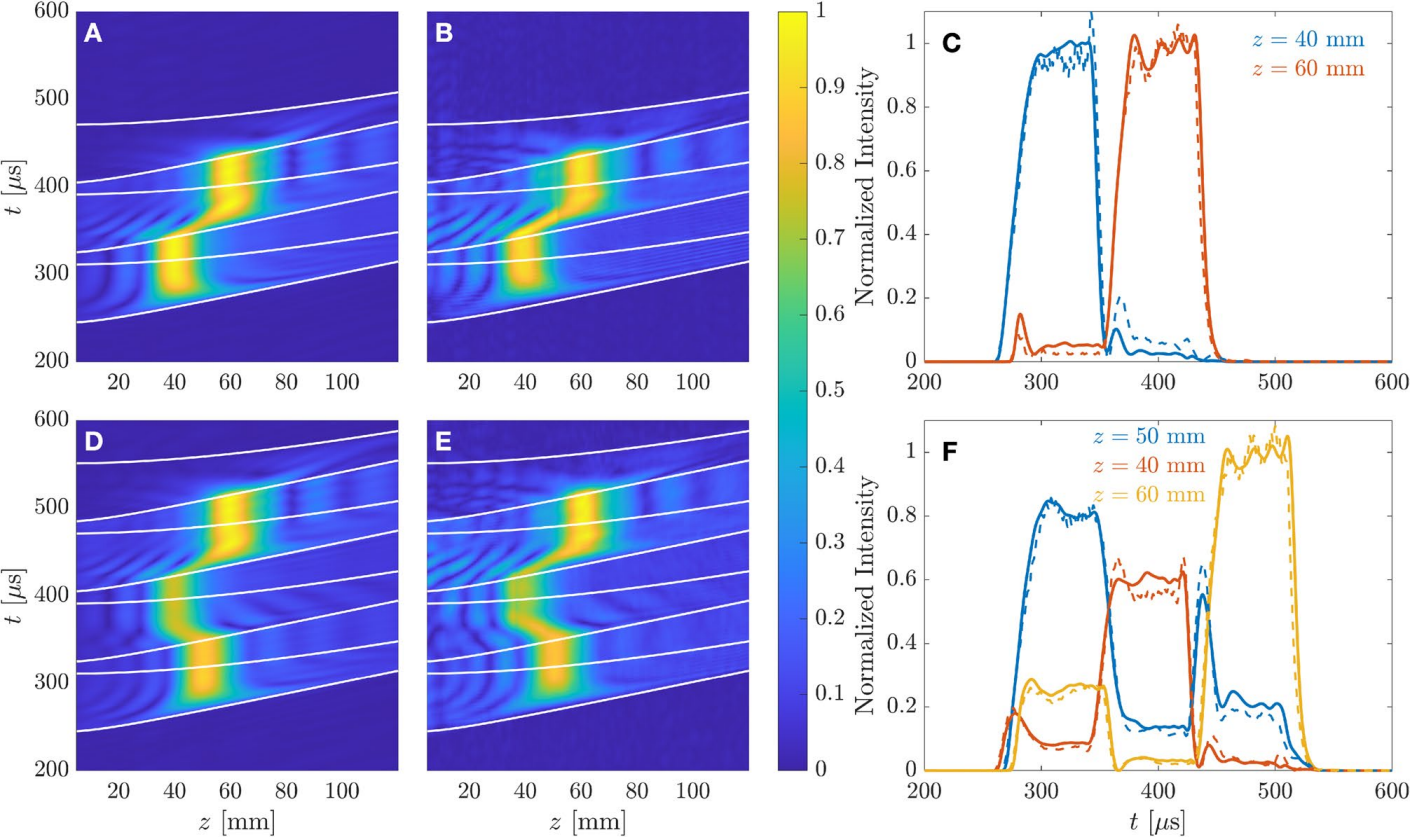
Simulated and measured results for the two waveforms depicted in Fig. 1C,D: (**A**) simulated and (**B**) measured pressure profile as a function of time using the two foci waveform, and (**C**) focal intensities as a function of time. (**D**) Simulated and (**E**) measured pressure profile as a function of time using the three foci waveform, and (**F**) focal intensities as a function of time. White lines in (**A**), (**B**), (**D**), and (**E**) represent the beginning and ending of transient states. In (**C**) and (**F**), solid lines represent simulation results, while dashed lines are for measurements.

As can be observed from Fig. 1A, the lens presents a single main pressure focus located at its theoretical design focal distance of 50 mm when the operating frequency corresponds to the design frequency of 250 kHz, which is shifted towards the lens when the frequency is reduced and away from the lens when the frequency is increased. Moreover, as can be seen in Fig. 1A, the maximum pressure value is achieved around the central frequency. Figure 1B depicts the focal distance and the normalized focal intensity as a function of the operating frequency. From Fig. 1B, the modulation bandwidth (BW) of the system can be calculated as the frequency span where the focal intensity is equal or higher than half of the maximum focal intensity value. Therefore, the system provides an experimental modulation BW of 85 kHz (34% respect to the central design frequency) ranging from 208 to 293 kHz, which results in an achievable focal distance range that goes from 33 to 68 mm (70% respect to the central FZP design focal distance). By using these results, it is possible to create an input waveform of contiguous rectangular pulses that can modulate the spatial response of the lens in the time domain, achieving an arbitrary number of foci and relative acoustic intensities. For this, first, the focal distances, normalized intensities, and time sequence of the foci are selected. Then, the pulse width has to be configured such that its duration is longer than the transient state duration and the steady response of the lens can be achieved^36^. Next, Fig. 1B is used to select the frequencies and amplitudes that provide the desired focal distances and normalized intensities. The pulse amplitude is calculated as $V_i=1/\sqrt{I_F(f_i)}$, being $I_F(f_i)$ the normalized focal intensity and $f_i$ the frequency that provides the design focal distance $F_i$. Finally, the waveform is formed by concatenating rectangular pulses with the calculated frequencies and amplitudes in the desired time order, and normalized so its maximum amplitude corresponds to the desired peak voltage ($V_p$). In this sense, Fig. 1C represents an example of a waveform designed to achieve focal distances of $F=\{40, 60\}$ mm with normalized focal intensities of $I_F=\{1, 1\}$, whereas Fig. 1D depicts a waveform calculated for $F=\{50, 40, 60\}$ mm and $I_F=\{0.8, 0.6, 1\}$. The pulse duration in Fig. 1C,D is set to 80 $\upmu$s, which ensures reaching a steady lens response. These waveforms would allow focusing an ultrasound beam at two and three focal distances within a single scan, respectively. However, it is worth noting that one drawback of this method is that it does not allow to achieve all the foci at the same time using a single continuous wave with all the required frequency components added simultaneously, due to the phase interference pattern among the different complex pressure profiles of each frequency (see “Supplementary Information” for more details).

One important parameter of every acoustic focusing system is its spatial resolution in both axial (perpendicular to the lens) and lateral (parallel to the lens) directions. In this sense, the proposed spatio-temporal modulation technique does not allow to control the resolution of the system by shifting the operating frequency, in contrast to the focal distance and focal intensity. Therefore, once the FZP is manufactured, the resolution of the system is constrained, meaning that, for a given focal distance, the resolution is fixed and cannot be controlled independently from the focal distance. However, due to the diffractive nature of FZPs, increasing the size of the lens, and therefore the number of Fresnel regions, will provide a focus with higher resolution. Thus, the number of Fresnel regions has to be selected according to the spatial resolution specifications required for each particular application. In this case, the systems achieves an experimental lateral resolution of approximately 3.5 mm, for the full frequency range, while the axial resolution ranges from 12 to 18 mm (see “Supplementary Information” for more details).

Figure 2 depicts the experimental results measured using the waveforms represented in Fig. 1C,D, compared to ideal numerical simulations computed using Eq. (3), showing very good agreement between simulations and experiments. The measured pressure profiles have been calculated as the absolute value of the complex Hilbert transform of the measured signal, for each one of the scanning positions along the central axis of the lens. Figure 2A,B represent the simulated and measured profiles as a function of time for the two foci waveform case, respectively, while Fig. 2C depicts the focal intensities as a function of time for each focus. Time spans in Fig. 2 start at 200 $\upmu$s because the propagation delay from the transducer to the shortest focal distance ($F=40$ mm) is 262.41 $\upmu$s. As can be observed in Figs. 2A,B, the pressure profile presents an initial transient state, depicted between the first two white lines (in time order), and then a steady response with a focus located at the first design focal distance ($F=40$ mm). As mentioned before, this transient response is a consequence of the planar shape of the lens, which results in diffracted pressure pulses with longer propagation paths for the outer Fresnel regions than for the inner regions, and therefore the lens only achieves its steady response once the pressure contributions from all regions overlap at the focus. Next, once the first rectangular pulse ends after 80 $\upmu$s and starts the second pulse designed to focus at $F=60$ mm, the pressure profile presents another transient state, depicted between the third and fourth white lines, where the pressure focus shifts from the first to the second focal distance. Then, the lens achieves a steady focus at its designed second focal distance ($F=60$ mm), followed by one last transient state depicted between the fifth and sixth white lines. Therefore, the transition state duration from one focus to the next is directly given by the corresponding transient state duration. As shown in Fig. 2C, both foci reach approximately the same intensity level, which agrees with the design goal. Analogously, Fig. 2D–F show the results for the three foci waveform case. As expected, the lens first focuses the ultrasound beam at $F=50$ mm with a normalized intensity level of $I_F\approx 0.8$, then shifts to $F=40$ mm with $I_F\approx 0.6$, and finally focuses at $F=60$ mm with $I_F\approx 1$. However, some cross-talk among the different foci can be observed in Fig. 2F, due to the limited axial resolution. This means that, for instance, the $F=50$ mm focus presents an axial spread wide enough to generate intensity signal at both $F=40$ and $F=60$ mm foci.

**Figure 3:**
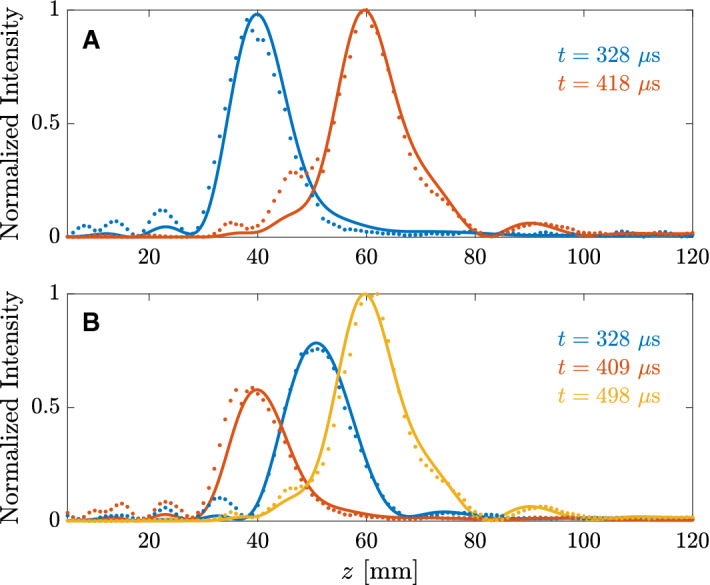
Steady focusing profiles: (**A**) two foci waveform and (**B**) three foci waveform. Solid lines represent simulation results, while dots represent experimental measurements.

Figure 3 depicts the steady intensity profiles compared to their ideal simulations, showing again good agreement between experiments and numerical calculations. As can be seen from the results, both waveforms depicted in Fig. 1C,D achieve their corresponding design goal in terms of both focal distance and normalized intensity. The measured axial resolutions for the 40, 50, and 60 mm foci are 12.81, 14.11, and 13.02 mm, respectively.

## Discussion

In summary, the proposed design method allows to modulate in space and time the response of a conventional monofocal FZP, achieving multiple ultrasound foci with arbitrary focal intensities. Experimental measurements and numerical simulations have been presented for two different case examples, one with two equal intensity foci and other with three foci with different acoustic intensities, demonstrating the potential of the proposed technique.

As mentioned earlier, due to the diffractive nature of the lens, once the lens is manufactured, the resolution is fixed and cannot be dynamically tuned for each focal distance. Therefore, if a higher axial or lateral resolution is required for a particular application, the number of Fresnel regions of the FZP should be increased. Alternatively, to increase the spatial resolution of the system while reducing its size, a higher frequency range could be employed, as the spatial properties (size and resolution) of the system are proportional to the central design wavelength. The drawbacks of increasing the frequency are mainly associated to the fact that RF electronics become more complicated and to the fact that higher accuracy and spatial resolution are required to manufacture the lens. In addition, ultrasound attenuation increases when using higher frequencies, which can be limiting in some biomedical scenarios. Other limitation of the proposed spatio-temporal modulation technique is the achievable focal distance range, which is limited by the modulation BW. In this sense, the modulation BW is limited by the combination of two factors: the BW of the transducer and the distortion introduced by the FZP itself, as a consequence of operating the lens at a frequency different from its original design frequency. Therefore, to increase the modulation BW, a transducer with a higher BW could be employed, or an FZP with a lower number of Fresnel regions (at the expense of reducing the spatial resolution of the system).

The method can be implemented in current ultrasound focusing systems where FZP lenses are employed, at a low cost, as only an AWG is required to generate the input waveforms. Although the proposed method is not able to focus simultaneously at all the focal distances, it is worth keeping in mind that the duration and amplitudes of each focus can be controlled individually, and even the delay between pulses could be tuned to provide specific target pressure profiles. This has potential applications in non-destructive testing or medical imaging where multiple focal planes could be scanned within one single pulse. In addition, the method could be used in medical ultrasound scenarios, creating ablation patterns with different tissue depths, allowing to specify individual ablation times and intensities for each target area.

## Materials and methods

### Experimental set-up

**Figure 4:**
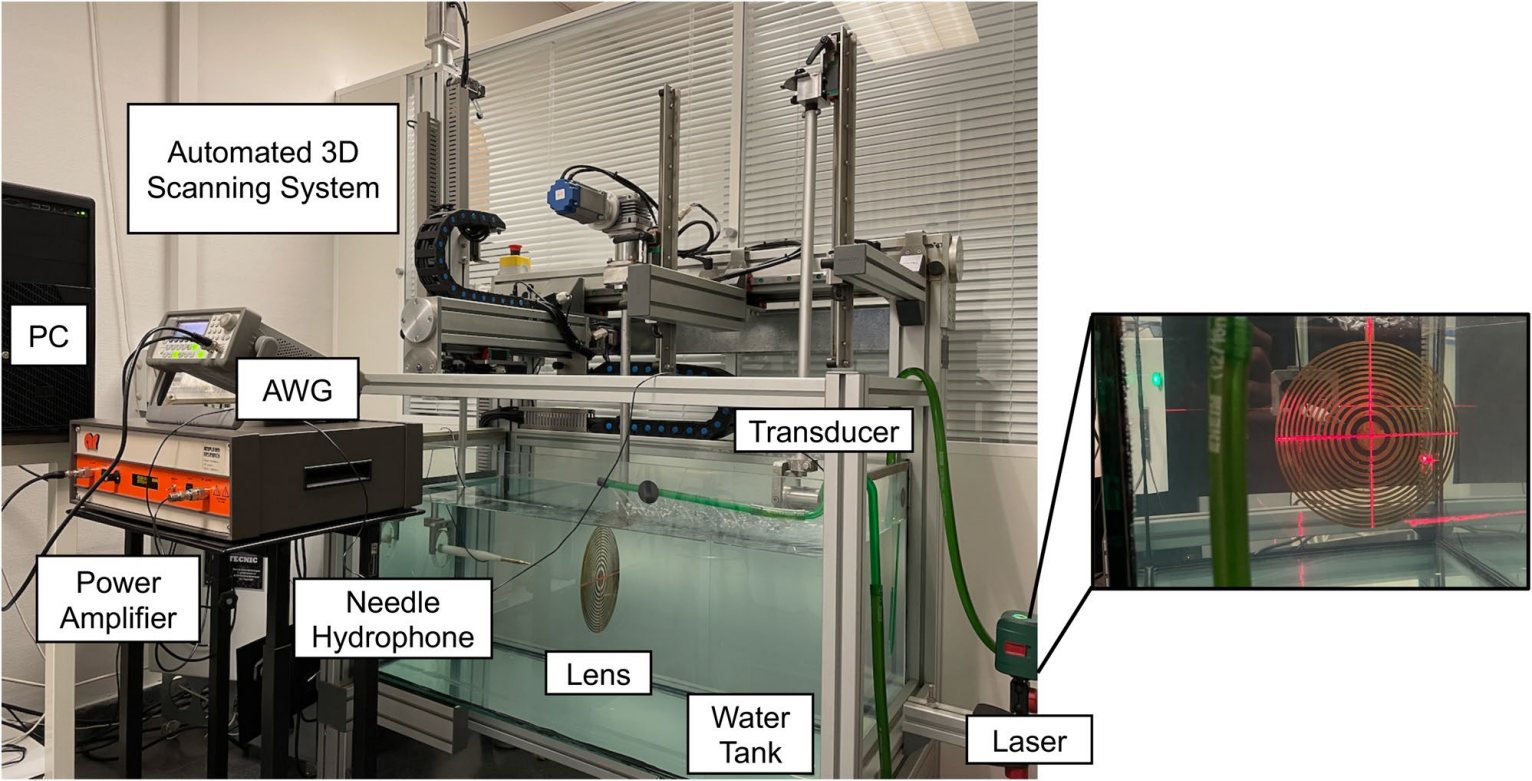
Picture of the experimental set-up (left side) and inset with a picture of the system position and level calibration using an self-leveling laser device (right side).

The experimental set-up consists of a 1 mm needle hydrophone from Precision Acoustics Ltd. attached to a robotic arm that can move along the three spatial axis with a resolution of $1\times 1\times 1$ mm$^3$. The robotic arm is connected to an automated positioning system controlled by a PC with a National Instruments NI7330 controller card and a custom-made LabView software that automatically controls the measurement and scanning process. The signal is digitized using a PicoScope 3224 USB digital oscilloscope. The transmitted signal is generated using an Agilent 33220A Arbitrary Waveform Generator (AWG) controlled by a PC with MATLAB R2018a (MathWorks Inc.). The output of the AWG is connected to a 75 W power amplifier 75A250A from Amplifier Research, and then to a 30 mm aperture immersion transducer from Imasonic, with a central frequency of 250 kHz. Figure 4 shows a picture of the experimental set-up. Before starting the measurements, a cross-shape self-leveling laser is used to spatially align the hydrophone with the lens and the transducer, as depicted in the right side of Fig. 4.

### Axial spectrum simulation

The axial spectrum of a FZP, given by $|p_z(\omega ,z)|$, can be calculated using the Rayleigh-Sommerfeld diffraction integral^37^ evaluated at the central axis of the lens, which eliminates the azimuthal dependence of the 2D integral over the lens surface due to its rotational symmetry and yields to the simplified expression

3 $$\begin{aligned} p_z(\omega ,z) = -jkX(\omega )z \int _0^{+\infty } p_i(\rho )t(\rho ) \frac{e^{jk\sqrt{\rho ^2+z^2}}}{ \rho ^2+z^2 } d\rho , \end{aligned}$$

where $X(\omega )$ is the input waveform spectrum, $k=\omega /c_0$ is the wavenumber, $p_i(\rho )$ is the incident pressure at the lens, $t(\rho )$ is the transmittance or pupil function of the lens (which is 1 at the transparent regions and 0 at the pressure blocking regions), and $\rho$ is the radial axis along the surface of the lens. Once the axial spectrum is calculated for each input waveform, the transient response of the lens is calculated as the inverse Fourier transform of the spectrum, $p_z(t,z)=|{\mathscr {F}}_\omega ^{-1}\{p_z(\omega ,z)\}|$.

## Supplementary information

Supplementary Information.

Supplementary Video 1.

Supplementary Video 2.

## Data Availability

the data that support the findings of this study are available from the corresponding author upon reasonable request.
