# Supplementary material for: Spatio-temporal ultrasound beam modulation to sequentially achieve multiple foci with a single planar monofocal lens
Source: Sci Rep. 2021 Jun 29;11:13458. doi: 10.1038/s41598-021-92849-x (PMC8242085; doi:10.1038/s41598-021-92849-x)
Supplement: Supplementary file 1 — Supplementary Information. [file 41598_2021_92849_MOESM1_ESM.pdf]

Supplementary Information: Spatio-temporal ultrasound beam modulation to sequentially achieve multiple foci with a single planar monofocal lens

Sergio Pérez-López[1], José Miguel Fuster[1], and Pilar Candelas[1,*]

[1]Centro de Tecnologías Físicas, Universitat Politècnica de València, 46022 Valencia, Spain
[*]Correspondence: pcandelas@fis.upv.es

May 21, 2021

Simultaneous multifocal simulations

One of the main drawbacks of the proposed method is that, although it can provide multiple foci, it cannot provide them simultaneously. This is due to the intrinsic nature of the complex frequency response of the lens. One could think that, for instance, the resulting focusing profile of two simultaneous signals with different frequencies added together would result in the combination of the two corresponding intensity focusing profiles for each frequency. But this is not possible, as the combination of both focusing profiles is not calculated using the intensity profiles but the complex pressure profiles, and therefore the phase response of both profiles interfere with each other resulting in a time-varying intensity distribution. Thus, in the steady state each profile can be described as $p_n(z,t) = p_n(z)e^{j\omega_n t}$, being $p_n(z)$ the absolute axial pressure distribution and $e^{j\omega_n t}$ the harmonic time dependent term. Let's consider the case of a $p(z,t)$ complex pressure distribution made from the combination of two profiles with different frequencies. The norm of such pressure profile can be expressed as

$$\begin{aligned} |p(z,t)| &= |p_1(z,t) + p_2(z,t)| = |p_1(z)e^{j\omega_1 t} + p_2(z)e^{j\omega_2 t}| \\ &= \sqrt{p_1(z)^2 + p_2(z)^2 + 2p_1(z)p_2(z)\cos\left((\omega_2-\omega_1)t\right)}, \end{aligned} \tag{1}$$

which presents a time dependent term with frequency $\omega_2 - \omega_1$ that will result in a time varying pressure profile, therefore highly distorting the target pressure distribution. To showcase this phenomenon, Figure S1 shows simulations of two waveforms using the same frequencies and amplitudes as those calculated in the main manuscript to achieve two equal intensity foci ($F = \{40, 60\}$ mm and $I_F = \{1, 1\}$) and three foci with different amplitudes ($F = \{40, 50, 60\}$ mm and $I_F = \{0.6, 0.8, 1\}$), but using a waveform that simultaneously presents all the frequency and amplitude components. For this simulation, continuous wave excitation is considered, so only steady state responses are shown. As can be observed from the results, the focusing profiles are highly distorted and time dependent, and they do not provide the desired two and three foci configuration, due to the interference among the different frequency components. In contrast, the proposed method in the main manuscript, which employs contiguous rectangular pulses with the different frequencies and amplitudes, is able to achieve the desired multifocal pattern

very accurately. For instance, as can be shown in Figures S1(a) and S1(c), the pressure profile presents a periodic response with frequency $f_d = f_2 - f_1 = 275 - 225 = 50$ kHz that results in a periodic focusing pattern with $T_d = 1/f_d = 20\ \mu$s. Moreover, as can be observed, although the pressure profile presents two foci, the response is highly distorted and the foci do not achieve their target acoustic intensities. Analogously, Figures S1(b) and S1(d) represent the results of the three foci waveform, showing an even more distorted profile as a result of the more complex interference pattern among the three harmonic responses.

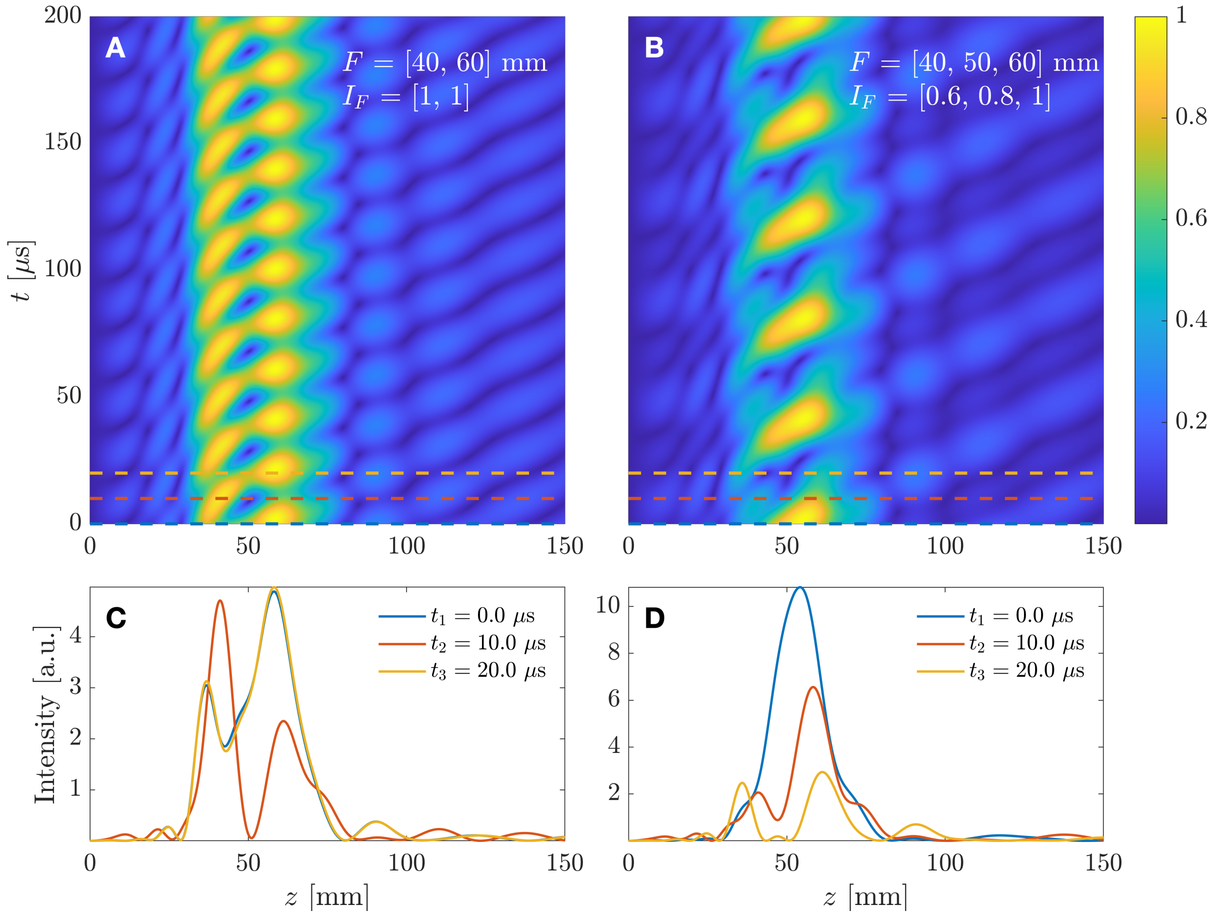

Figure S1: Continuous wave simulation results: **(A)** pressure profile as a function of time for two equal intensity foci at 40 and 60 mm, and **(B)** pressure profile for three foci at 50, 40, and 60 mm with normalized intensities 0.8, 0.6, and 1, respectively. Panels **(C)** and **(D)** represent axial cuts at three different times for the two foci and three foci cases, respectively.

Spatial resolution of the system

Spatial resolution is an important parameter of every acoustic focusing system. In this case, the spatial resolution of the system is given by the length and width of the acoustic intensity focus, specified by the FLHM (Full-Length Half Maximum) and FWHM (Full-Width Half Maximum), respectively. The FLHM is measured along the perpendicular axis of the lens (z-axis), while the FWHM is measured along the parallel axis to the lens (r-axis). Both values are calculated from the measured steady 2D intensity map, $|p(f, r, z)|^2$, for each operating frequency. As discussed in the main manuscript, once the lens is manufactured, the resolution of the system at a given focal distance cannot be controlled individually with the operating frequency. If a higher resolution is required for a particular application, the size of the lens or the central frequency of the

system should be increased. As can be observed from Figure S2, the measured lateral resolution remains almost constant at 3.5 mm in all the frequency range, while the axial resolution ranges from 12 to 18 mm. However, in terms of the operating wavelength, the resolution decreases when the frequency increases. For the three considered focal distances in the main manuscript, $F = \{40, 50, 60\}$ mm, the measured lateral resolutions are FWHM $= \{3.38, 3.49, 3.53\}$ mm while the measured axial resolutions are FLHM $= \{12.81, 14.11, 13.02\}$ mm, respectively.

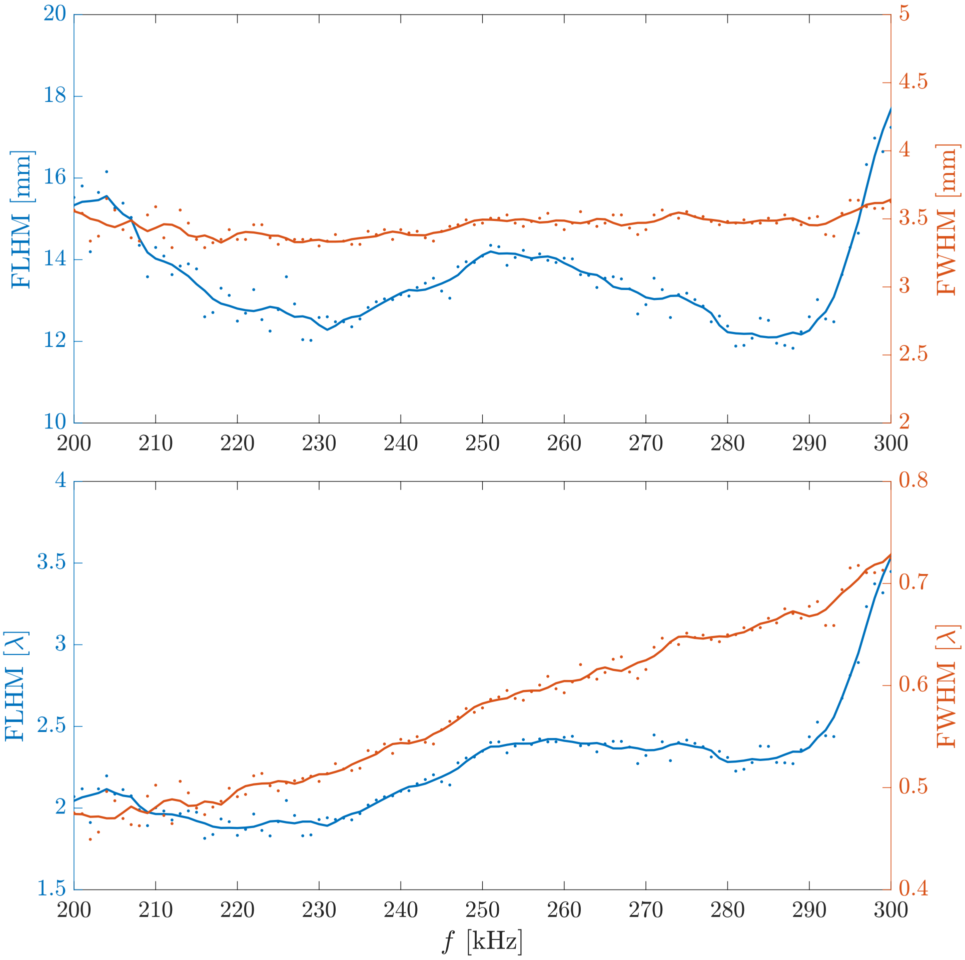

Figure S2: Measured axial (FLHM, left side) and lateral (FWHM, right side) resolutions as a function of the operating frequency. Top panel corresponds to absolute resolution values, while bottom panel corresponds to resolution values normalized to the operating wavelength.

Experimental axial spectrum characterization

The axial spectrum of the lens is measured using a three step process. First, the input waveform is designed and uploaded to the AWG directly using its associated MATLAB interface. Then, the axial profile is measured using the 3D automated positioning system described in the Materials and Methods section of the main manuscript, which provides the axial profile as a function of time. Finally, the axial spectrum is calculated as the Fourier transform of the measured time domain pressure profile, and then equalized using the spectrum of the input waveform to compensate its effect on the measured response, that is

$$p_z^{eq}(\omega, z) = \frac{p_z(\omega, z)}{|X(f)|}, \tag{2}$$

being $X(f)$ the spectrum of the input waveform.

The input pulse is designed to provide a gaussian spectrum centered at the central design frequency of the lens ($f_0 = 250$ kHz) with a bandwidth (Δf) wide enough to characterize the lens response at the design frequencies. In this case the bandwidth is set to $\Delta f = 100$ kHz, which allows to measure the axial spectrum from 200 to 300 kHz. The pulse is calculated as

$$x(t) = V_p \cdot \exp\left(-\frac{(t-t_0)^2}{2\sigma_t^2}\right) \cdot \sin\left(2\pi f_0 (t-t_0)\right), \tag{3}$$

where V_p is the peak voltage of the waveform, t_0 is the time point when the pulse envelope reaches its maximum value, and σ_t is the standard deviation of the pulse, given by

$$\sigma_t = \frac{\sqrt{\ln 4}}{\pi \Delta f}. \tag{4}$$

In this case, the pulse duration is set to 50 μs and t_0 is set to 25 μs. Figure S3(a) shows the input waveform and Figure S3(d) depicts its corresponding spectrum. Figure S3(b) represents the measured time domain bipolar pressure profile, while Figure S3(c) depicts the absolute value of its complex Hilbert transform along the time axis. Finally, Figure S3(e) represents the absolute value of the Fourier transform of Figure S3(b), and S3(f) depicts the axial spectrum after equalizing Figure S3(e) using the spectrum shown in Figure S3(d).

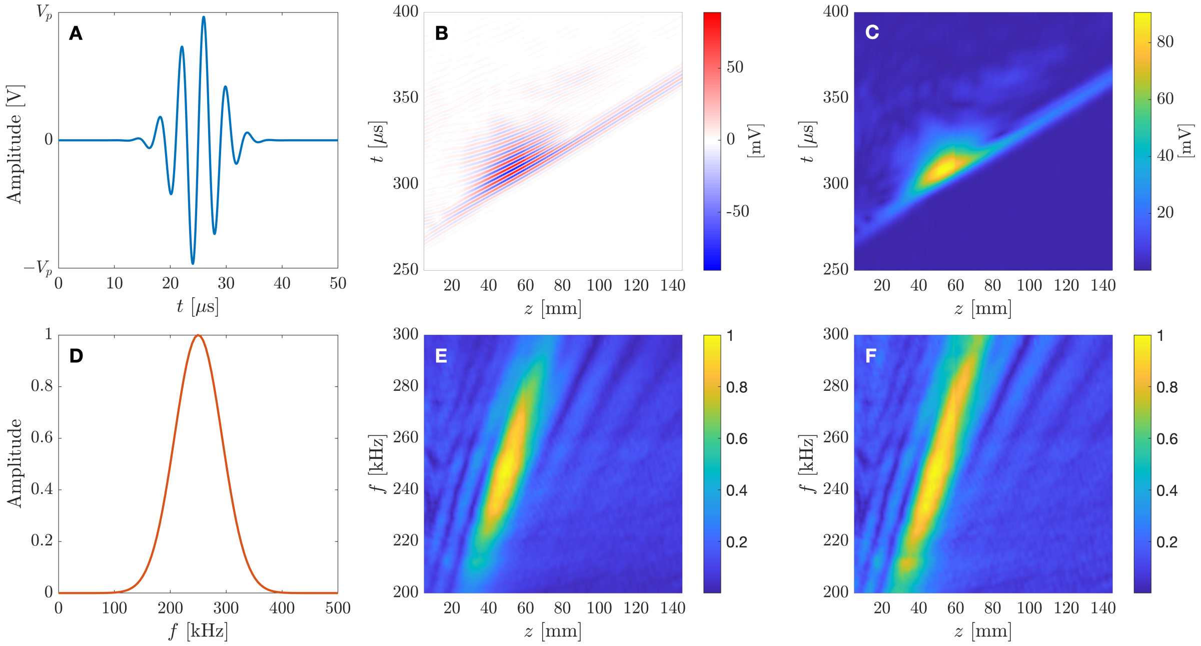

Figure S3: Axial spectrum characterization: **(A)** input modulated gaussian pulse used for the characterization and **(D)** its corresponding spectrum. **(B)** Measured bipolar pressure profile as a function of time and **(C)** absolute value of its complex Hilbert transform. **(E)** Absolute value of the measured pressure profile Fourier transform and **(F)** equalized axial spectrum of the lens.
